# Clinical Characteristics and Outcomes of Patients with Intracerebral Hemorrhage – A Feasibility Study on Romanian Patients

**DOI:** 10.25122/jml-2020-0042

**Published:** 2020

**Authors:** Razvan Alexandru Radu, Elena Oana Terecoasa, Cristina Tiu, Cristina Ghita, Larisa Irina Purcaru, Andreea Nicoleta Marinescu, Ovidiu Alexandru Bajenaru

**Affiliations:** 1. Department of Neurology, University Emergency Hospital, Bucharest, Romania; 2. “Carol Davila” University of Medicine and Pharmacy, Bucharest, Romania; 3. Department of Radiology and Medical Imaging, University Emergency Hospital, Bucharest, Romania

**Keywords:** ICH, ICH-Prognosis, ICH-volume, ICH-etiology, outcome, CAA = Cerebral amyloid angiopathy, GCS = Glasgow coma score, mRS = modified Rankin score, NIHSS = National Institute of Health Stroke Scale

## Abstract

Intracerebral hemorrhage is a significant public health problem, as it is a disease associated with overwhelming mortality and disability. We performed a retrospective feasibility study of patients admitted with acute intracerebral hemorrhage in our department for four months. Our aims were to identify peculiarities of the risk factors, demographic and clinical characteristics of intracerebral hemorrhage patients from our population, to estimate a feasible recruitment rate for a larger prospective study of patients with intracerebral hemorrhage and to analyze and correct potential drawbacks in the methodology of a more extensive prospective study of patients with intracerebral hemorrhage hospitalized in our department. During the study period, we admitted 53 patients with intracerebral hemorrhage in our department. The mean age of the patients was 69.1 years, and 53% were men. Arterial hypertension was the most common etiologic factor leading to intracerebral hemorrhage. 50.01% of patients died during hospitalization, 31.19% were discharged with significant disability, and 18.8% had a favorable short-term outcome. Higher hematoma volumes, male sex, deep location of the hemorrhage, and age between 51 and 60 years were factors associated with an unfavorable short-term outcome.

## Introduction

Stroke is considered an overwhelming global health problem [1]. In 2016, 10.1% of global deaths were caused by stroke, and there were 13.7 million new stroke cases worldwide with an overall cost of 116 million disability-adjusted life years [2,3]. Although the global incidence and mortality rates of stroke have decreased during the past 20 years, the absolute number of people suffering a stroke has almost doubled from 1990 to 2016, and the absolute number of stroke-related deaths increased by 20% over the same time period, stroke mortality being now ranked as the second leading cause of death, after ischemic heart disease [4,5]. Current estimates predict that without specific measures, the global burden of stroke will continue to increase during the next decades [1,6].

According to the Global Burden of Disease 2017 Study, 65% of the worldwide strokes are ischemic strokes, 26% are intracerebral hemorrhages (ICH), and 9% subarachnoid hemorrhages [7]. Despite having a much lower prevalence than ischemic stroke, ICH accounts for a significant proportion of the stroke-associated burden worldwide [8], as 48.4% of the stroke-related deaths and almost 20% of global stroke survivors are attributable to ICH [7].

The lack of an effective treatment for ICH makes the study of the specific features of the individuals most commonly affected by ICH of utmost importance. Reliable data regarding the particularities of risk factors, demographic, clinical, and imaging characteristics and most common etiologies for intracerebral hemorrhage in the Romanian population are sparse. Differences between ICH burden and outcomes in different regions of the world can be attributed to local differences regarding comorbidities, lifestyle, diet, economic status, and health system. In-depth knowledge regarding the local pattern of risk factors and demographic and clinical characteristics of patients with ICH could support the implementation of targeted prevention measures for this disease. This study aims to identify peculiarities of the risk factors, demographic and clinical characteristics of ICH patients from our population, to estimate a feasible recruitment rate for a larger prospective study of patients with ICH and to analyze and correct potential drawbacks in the methodology of a larger prospective study of patients with ICH hospitalized in our department.

## Material and Methods

We performed a retrospective cross-sectional study of patients with non-traumatic intracerebral hemorrhage identified by non-contrast computed tomography who were admitted in the Department of Neurology of the University Emergency Hospital Bucharest between January 2018 and May 2018. We included all patients with non-traumatic ICH regardless of etiology. The baseline clinical, demographic, and radiological data were extracted from the patients’ medical records, the hospital electronic database, and the hospital’s imaging database. Our hospital ethics committee approved this study, and participants gave informed consent.

Initial stroke severity was classified according to the National Institute of Health and Stroke Scale (NIHSS) as follows: mild stroke (NIHSS 0-5 points), moderate stroke (NIHSS 6-10 points), moderate-severe stroke (NIHSS 11-15 points), severe stroke (NIHSS 16-20 points), very severe stroke (NIHSS > 20 points). Short term ICH outcome was assessed with the modified Rankin Scale (mRS), and patients with mRS scores of 0 – 2 were considered functionally independent. Both scores were calculated by attending physicians during hospitalization.

Traditional risk factors were defined as follows:

1. diabetes mellitus - clinical history of diabetes mellitus or glycated hemoglobin ≥ 6.5% or random plasma glucose of ≥ 200mg/dl or fasting glucose level ≥ 126mg/dl;
2. dyslipidemia - low density lipoprotein cholesterol ≥ 100mg, triglycerides ≥ 150 mg/dl or previous treatment with statins;
3. arterial hypertension - history of elevated systolic blood pressure (≥ 140 mmHg) and/or diastolic blood pressure (90 mmHg);
4. coexistent cardiovascular disease - history of ischemic heart disease and/or diagnosis of ischemic heart disease during hospitalization;
5. chronic alcohol intake – more than 40 g/ethanol per day regularly. A previous stroke was defined as any past clinical history of stroke (silent lesions found on CT scans were not taken into consideration).

All patients underwent a brain CT scan at admission, and follow-up CT scans at predefined time intervals or in case of worsening of the clinical status. Hematoma location and presence of intraventricular bleeding were retrospectively assessed on the initial CT scan. Hematoma volumes were also retrospectively measured on initial CT scans by authors E.O.T and L.I.P using the ABC/2 method [9, 10]. Intraventricular bleeding was not included in the final hematoma volume calculation.

ICH etiology was retrospectively classified using the SMASH-U criteria [11] based on the medical information available in the medical records by authors E.O.T. and R.A.R. Cause of death was extracted from medical records by authors E.O.T. and L.I.P. and classified into a specific major group according to predefined criteria.

All statistical analyses were performed using the MedCalc Statistical Software version 18.11.3 (MedCalc Software, Ostend, Belgium) and NCSS 12 Statistical Software (NCSS, LLC. Kaysville, Utah, USA). Continuous variables are described as mean ± standard deviations (SD) and categorical variables are presented as absolute numbers and percentages. For comparisons between continuous variables, we used Mann – Whitney or Kruskal – Wallis tests, according to the number of selected groups analyzed. For testing the strength of association between categorical variables, we used the Chi-squared test or Fisher’s exact test, depending on the number of analyzed cases. Statistical analysis was limited to bivariate analysis due to the small sample size of this feasibility study. A preset significance level of p < 0.05 was used for all comparisons.

## Results

**Figure 1:**
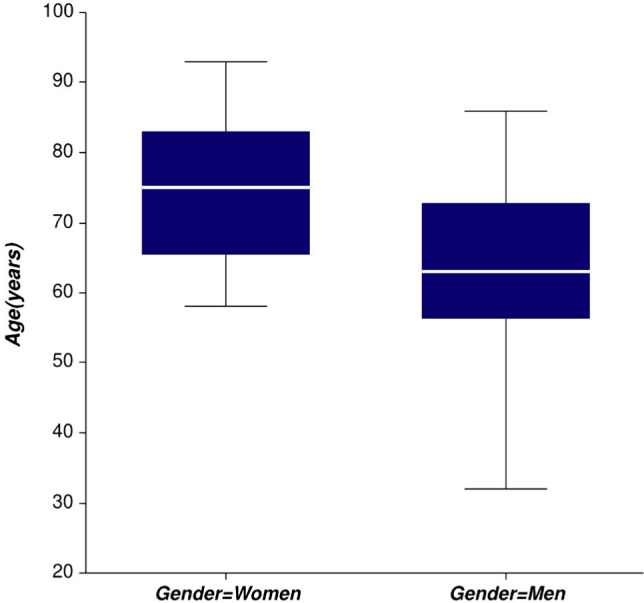
Median age according to gender in the study population.

**Figure 2:**
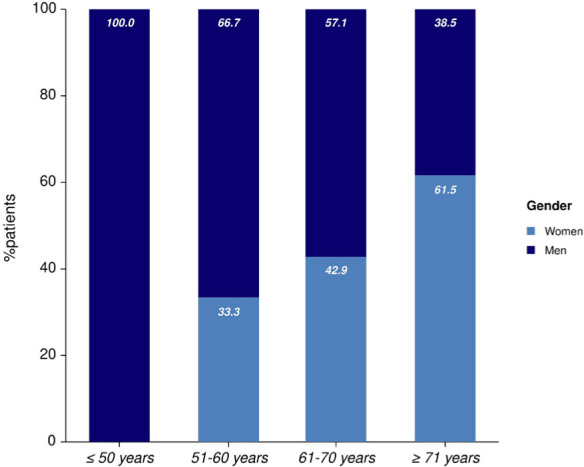
Gender distribution according to age categories.

During the analyzed period, 53 patients were admitted to the Neurology Department with the diagnosis of acute intracerebral hemorrhage. The general and baseline characteristics of the patients are presented in Table 1. The mean age of the patients was 69.1 years ± 12.5 years, and 53% were males. Women admitted with ICH were significantly older than men (median age was 74.28 vs. 64.46 years, p= 0.003, Figures 1 and 2). 7.55% of the patients were younger than 50 years, 16.9% were aged 51-60 years, 26.42% were aged 61-70 years, and 49.06% were older than 71. Out of the thirteen patients who suffered an ICH before the age of 60, 10 patients were men (76.9%).

**Table 1:** Baseline characteristics.

**Demographic**	
Age (years), mean ± SD	69.1 ± 12.5
Female Sex	25 (47%)
**Comorbidities and risk factors, n(%)**	
Arterial Hypertension	45 (84.91%)
Diabetes Mellitus	10 (18.8%)
Current Smoking	9 (16.98%)
Dyslipidemia	30 (56.6%)
Ischemic heart disease	9 (16.9%)
Previous stroke	9 (16.9%)
Chronic alcohol intake	13 (24.52%)
**Previous medication, n (%)**	
Previous antiplatelet treatment	10 (18.87%)
Previous anticoagulant treatment	6 (11.54%)
Previous antihypertensive treatment	19 (35.85%)
**Stroke severity and pre–stroke status**	
Pre-stroke mRS score, median	0
Admission Glasgow Coma Score, median	15
NIHSS, median	14
**Imaging**	
Lobar hematoma	18 (34%)
Deep hematoma	32 (60%)
Thalamic	14 (26%)
Putaminal	16 (30%)
Caudate	2 (4%)
Brainstem+ Cerebellar	3 (6%)
ICH volume (mL), median	10
Intraventricular effraction	20 (38%)

The prevalence of traditional vascular risk factors in our study population is detailed in Table 1. Arterial hypertension was the most common identified risk factor (84.91%) and was more common in women than in men (96% vs. 75%). 18.8% of patients had diabetes mellitus, which was more common in men than in women (25.9% vs. 12.5%). 56.6% of patients were diagnosed with dyslipidemia, which was more common in women than in men (76.6% vs. 66.6%).

**Figure 3:**
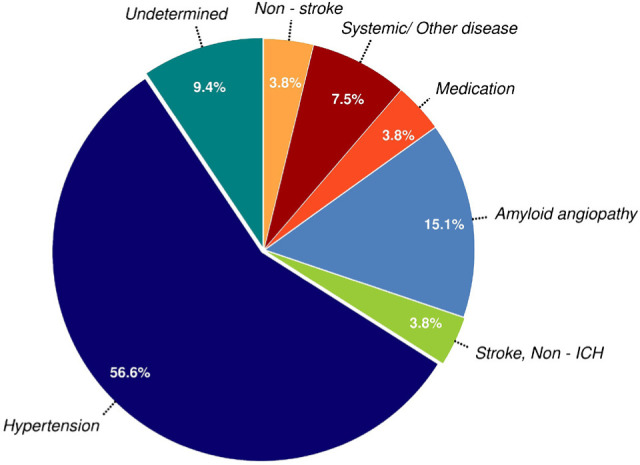
ICH etiology according to the SMASH-U criteria in the study population.

The most common ICH etiologies, as defined by the SMASH-U criteria, were: arterial hypertension (56.5%), possible amyloid angiopathy (15.1%), undetermined etiology (9.4%), systemic diseases (7.55%) and anticoagulation-related (3.77%). Four patients had ICH due to massive hemorrhagic transformation of an ischemic stroke and/or massive ICH following thrombolytic therapy (Figure 3). In our study population, ICH due to possible amyloid angiopathy was more frequently identified in women than men (75% vs. 25%), while there were no gender differences in the prevalence of ICH attributed to arterial hypertension (50% vs. 50%).

The majority of patients were in good-health before ICH onset as the median pre-admission mRS was 0, and 81.82% had a pre-stroke mRS of 0 or 1. 30.41% of patients were taking antiplatelet medication and 11.54% oral anticoagulants at the time of ICH. Even though 67.93% of the patients had a known history of arterial hypertension, only 35.85% regularly took antihypertensive medication at home.

**Figure 4:**
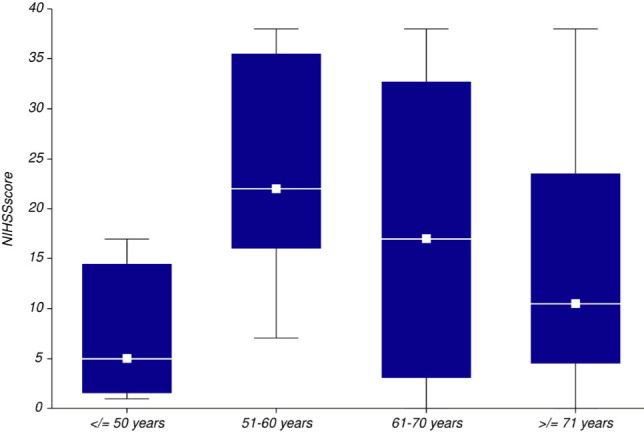
Initial stroke severity according to age groups in the study population.

The median Glasgow Coma Score at admission was 15 points (25-75 IQR 9-15 points). 15.69% of the patients had massive ICH and consequently, a GCS score of 3 points at admission. According to the NIHSS scale, initial stroke severity was mild for 30.19% of patients, moderate for 11.32%, moderate-severe for 11.32%, severe for 9.44%, and very severe for 37.74% of patients. Median NIHSS score at admission was 14 points, 50% of patients having NIHSS scores between 4 and 25 points. Stroke severity was significantly higher in men than in women (NIHSS score 21 vs. 7 points). The most severe strokes were observed in the 51- 60 years (median NIHSS score 22 points) and 61 - 70 years (median NIHSS score 17 points) age groups while patients younger than 50 years had a median NIHSS score of 5 points and patients over 71 years had a median NIHSS score of 10.5 points (Figure 4).

**Figure 5:**
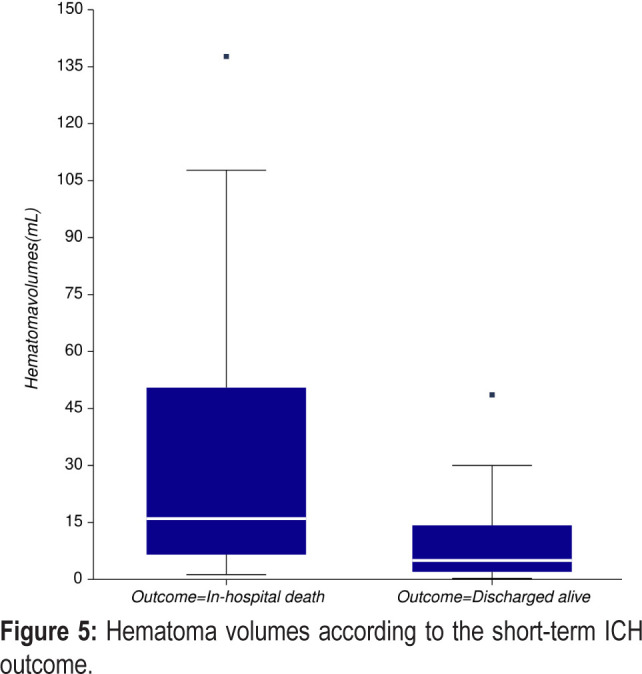
Hematoma volumes according to the short-term ICH outcome.

ICH location was at the basal ganglia level in 60% of cases, lobar in 34% of cases, and infratentorial in 6% of cases. 50% of the deep hematomas involved the lenticular nucleus, 44% involved the thalamus, and 6% the head of the caudate nucleus. Ventricular extension occurred in 38% of cases. As expected, lobar ICH was more common in patients older than 71 years and deep hemorrhages in patients younger than 61. The median initial hematoma volume for our study population was 10 ml, and 50% of the patients had blood volumes between 3.9 ml and 34.6 ml. There was a trend for higher hematoma volumes in men compared to women (12.7 ml vs. 6 ml, p= 0.06). However, despite this general trend, the highest hematoma volumes were observed in women aged 61 – 70 years (median 48.6 mL, Table 2). In terms of age, higher hematoma volumes were observed in patients aged 61-70 years (median 41.9 mL) and 51 – 60 years (median 16.5 mL). As expected, hematoma volumes were significantly associated with in-hospital death (15.9 ml vs. 4.9 ml, p=0.004, Figure 5) and were higher in patients in which the cause of death was considered to be the severity of ICH than in those with other presumed causes of death (median volumes 24.9 ml vs. 6.85 ml, p=0.03).

**Table 2:** ICH volumes for men and women of different age groups in the study population.

	**Median ICH volumes**		
	**General**	**Women**	**Men**
**≤** **50 years**	7.9 mL	-	7.9 mL
**51 – 60 years**	16.5 mL	5.75 mL	24.95 mL
**61 – 70 years**	41.9 mL	48.6 mL	28.3 mL
**≥ 71 years**	4.9 mL	3.95 mL	9. 75 mL

**Figure 6:**
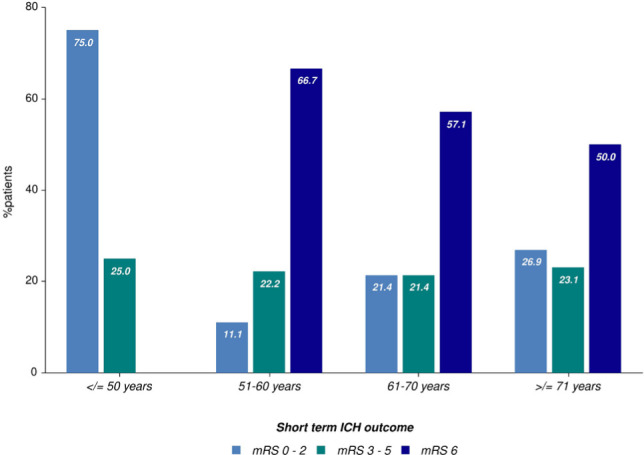
Short term ICH outcome according to age groups for the study population.

**Figure 7:**
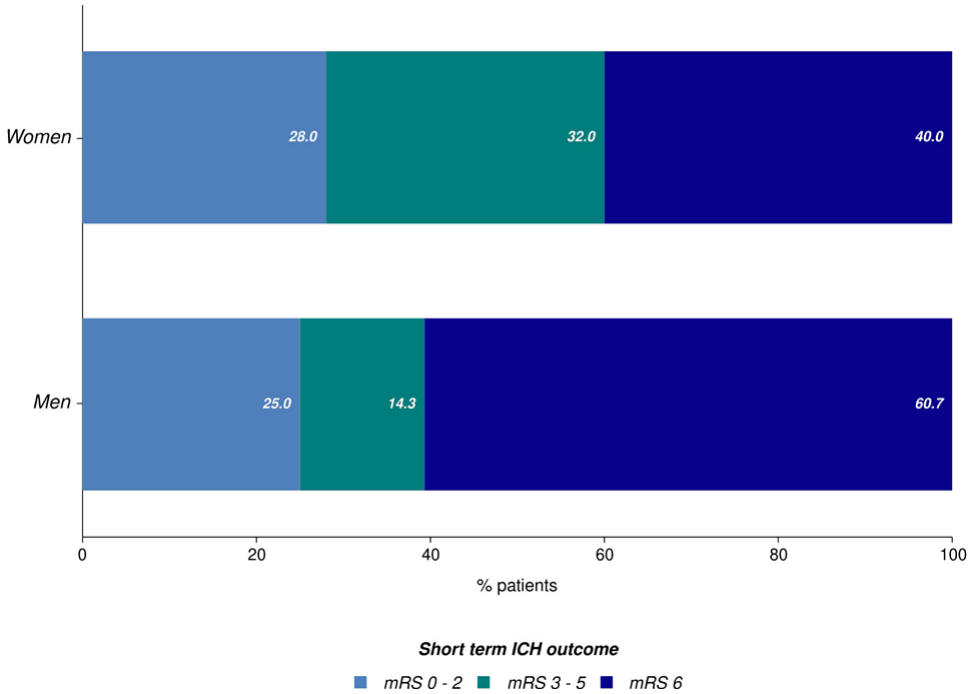
ICH outcome according to gender for the study population.

The in-hospital mortality was 50.1%. Age was not a significant predictor of in-hospital mortality, as the median ages of those who survived and those who died during hospitalization were similar (67.8 vs. 70.2 years p=0.4). 66.7% of the in-hospital deaths were directly attributed to ICH, 22.2% were considered to be the consequence of systemic infectious complications, and 7.4% were related to other coexistent diseases or complications (like major upper gastrointestinal bleeding, acute coronary syndrome or pulmonary embolism). Trends of in-hospital mortality between different age groups are worth to be mentioned. All patients younger than 50 years survived, whereas 67% of the patients aged between 51-60 years died during hospitalization (Figure 6). Mortality was significantly higher in men than in women (61% vs. 40%, Figure 7). Patients with ICH due to possible amyloid angiopathy had the lowest in-hospital mortality (13%) rate, whereas patients with ICH attributed to arterial hypertension had an in-hospital mortality rate of 47%. All patients with a GCS score of 3 points at admission died during hospitalization. Similarly, all patients with infratentorial hemorrhages died during hospitalization, but the small number of cases included in the study hampered the analysis of this subgroup. Lobar and thalamic hemorrhages had the lowest mortality rates (44.4% and 42.9%, respectively). 56% of the patients with deep hematomas died because of the severity of ICH, while 37% died due to systemic infectious complications. 26.3% of the patients were functionally independent at discharge (mRS scores 0-2), and 20.7% were discharged with severe neurological deficits (mRS scores 4 and 5).

## Discussion

The current study was a small retrospective feasibility study of patients with ICH. The study was not designed to draw definite conclusions regarding the peculiarities of the most common risk factors for ICH and demographic and clinical characteristics of patients with ICH from the local population but rather to highlight some hypotheses that are planned to be tested in a future prospective analysis of a larger cohort of Romanian patients with ICH. During four months, we admitted to our department 53 patients with ICH. Thus, assuming a constant admission rate of approximately 15 patients with ICH/month, a study designed to include 100 patients should have a recruitment period of at least seven months. However, it should be noted that robust data regarding the socio-demographic characteristics of patients with ICH cannot be obtained in all cases because sometimes the impact of the severity of the clinical status of patients with ICH on relatives can hamper further discussions regarding prior treatment, clinical and social status. Furthermore, a significant proportion of our patients are living alone, and the families cannot provide precise details regarding prior medical history and therapeutic schemes.

Hypertension is the most important risk factor for spontaneous ICH and the contribution of hypertension is higher for deep ICH than for lobar ICH [12]. In our study, 84.9% of patients with ICH suffered from hypertension (69.8% had a known history of hypertension, and 15.1% were diagnosed during hospitalization). There were no significant differences regarding the prevalence of arterial hypertension in patients with different ICH locations and ICH etiologies. Robust data regarding the prevalence of arterial hypertension in our population of patients with ICH should be obtained from a study that included a large number of patients. However, it may be reasonable to presume that trends in the Romanian population follow the ones from other countries. Unfortunately, worldwide, the absolute numbers of patients with arterial hypertension are continuously increasing due to the aging of the populations. However, the global percentage of those correctly treated has not improved dramatically due to a myriad of factors, including inconsistent health policies, poor awareness of the population, and poverty [13]. In our study, only 51.3% of the patients with ICH previously diagnosed with hypertension were taking antihypertensive drugs at home. If this disastrous percentage is to be confirmed in a larger population of patients, a further analysis of the reasons underlying this observation would be needed to design targeted national health policies.

Approximately one-third of our ICH patients were previously treated with antithrombotic drugs (18.8% with antiplatelets and 11.5% with anticoagulants). As the populations are aging and traditional vascular risk factors become more frequent, the number of patients suffering an intracerebral hemorrhage under chronic treatment with antithrombotic drugs is growing [14, 15]. It is still unclear if the effect of prior antiplatelet medication significantly influences ICH severity and outcome and, similarly, the different consequences of antithrombotic drugs on ICH attributed to hypertensive arteriolopathy and cerebral amyloid angiopathy are not entirely characterized yet [16]. Further details regarding differences in outcome between different ICH etiologies in our population previously treated with antithrombotic drugs need to be tested in a larger cohort.

Age is considered a predictor of increased mortality and is one of the main predictors of mortality in the ICH score [17]. However, in our study, the highest in-hospital mortality rate was observed among patients between 51 and 60 years old (66.7%) and the lowest among patients over 71 years (50%). This finding needs to be verified in multivariate analysis models, including different potential confounding factors like gender, comorbidities, ICH volume, etiology, and location. It is worth mentioning that in Romania, unlike other countries in Europe, do-not-resuscitate orders and withdrawal of care for the very severe cases of ICH is not a standard practice due to legislation so that this issue could explain small differences regarding short term ICH outcome between our cohort and international databases. Studies from countries in which withdrawal of care is a common practice for those considered to have no chance of survival might report higher mortality rates for elderly patients and patients with very severe ICH forms. Patients with ICH treated with full medical support for at least five days were shown to have a 30% less mortality than initially predicted [18]. Thus, further studies focusing on ICH mortality should take into account self-fulfilling prophecy bias resulting from withdrawal of care practices which may greatly differ between study sites and countries.

Stroke is an important cause of in-hospital mortality. However, determining the direct cause of death in stroke patients is not as straightforward as it seems, and accurate and reproducible determination of the cause of death is currently questionable [19]. Clinicians do not usually receive formal training to establish the cause of death, and ICD-10 rules are complex and not well understood [20. Patients with ICH sometimes have severe comorbidities or develop different systemic complications during hospitalization, which might also be life-threatening. Therefore, in these cases, it is debatable whether the main factor leading to death is the intracerebral hemorrhage or another worsening pre-existing condition or newly-developed disease triggered by the cascade of events accompanying the clinical course of a hemorrhagic stroke. This is why a future study aiming to establish the leading cause of death in stroke patients should provide a formal rating strategy and intra- and inter-rater agreement measurements.

Currently, available datasets regarding gender differences in the outcomes of patients with ICH yield conflicting results [21, 22]. A recent multi-ethnic analysis of the US population showed no significant interaction of gender and age on the outcome [23], unlike other studies proving that female gender may be an independent predictor of early mortality in patients with hemorrhagic stroke, even after adjustment for stroke severity, hematoma volume, and age [22]. In our study, in-hospital mortality was greater in men than women (60.7% vs. 40%), but the difference was not statistically significant (p=0.13). We consider that this finding will be confirmed in a future study with a larger sample size since we also observed a general trend for higher hematoma volumes in men compared to women (median volumes 12.7 mL versus 6 mL, p=0.06).

ICH volume is one of the main determinants of outcome in ICH patients [24]. Several hematoma volume cut-offs were proposed overtime in the literature to discriminate between a good and a bad outcome. However, it is now clear that hematoma volumes exceeding 30 mL are associated with an increased risk of hematoma expansion and in-hospital death [25, 26]. In our study, 29.4% of patients had hematoma volumes ≥ 30 mL, and 73.3% died during hospitalization.

Although the percentage of in-hospital deaths among this group was higher than for the group of patients with hematoma volumes < 30 mL (44.4%), the difference did not reach statistical significance (p=0.07), probably due to the small sample size. Whether this assumption regarding the 30 mL cut-off hematoma volume as a strong predictor of short-term ICH outcome is also applicable to the Romanian patients remains to be proven in a further study.

The ABC/2 method was shown to be sufficiently accurate to measure the hematoma volume, but it performs less well when the clot is large and irregular [27]. In our study, the ABC/2 volume was calculated by neurologists on unformatted CT images. Testing the accuracy of this approach in comparison with other methods of hematoma volume calculation, as well as the inter-agreement with radiologists, should be further investigated.

## Conclusions

The current study’s findings emphasized several methodological issues that have to be discussed before designing a feasible future prospective study on patients with ICH hospitalized in our department. Age, gender, and specific comorbidities seem to be strongly associated with the short-term outcome of patients with ICH, but their real relevance remains to be established in a future study. Special attention should be paid to the analysis of ICH patients aged between 51 and 60 years who appear to have an unusually high in-hospital mortality rate compared with patients of other ages. As opposed to the findings of similar studies from other countries, the male gender seems to be more frequently associated with a bad short-term outcome in our population of patients. Further evaluation of the clinical and demographic characteristics of Romanian patients with ICH and the analysis of specific traditional, social and cultural risk factors associated with bad outcomes could lead to the implementation of targeted primary ICH prevention strategies aiming to reduce the number of ICH attributable deaths in Romania.

## Conflict of Interest

The authors declare that there is no conflict of interest.

## Acknowledgment

The publishing of this article was supported by the Foundation of the Romanian Society of Neurology.
